# Opioid Overdose-Related Emergency Department Visits Pre- and Post-COVID-19

**DOI:** 10.5811/westjem.53227

**Published:** 2026-06-10

**Authors:** Melanie F. Molina, Samuel D. Pimentel, Maria C. Raven, Kathy T. LeSaint, David G. Dillon

**Affiliations:** * University of California, San Francisco, Department of Emergency Medicine, San Francisco, California; † University of California, San Francisco, Philip R. Lee Institute for Health Policy Studies, San Francisco, California; ‡ University of California, Berkeley, Department of Statistics, Berkeley, California; § University of California, Davis, Department of Emergency Medicine, Sacramento, California

## Abstract

**Introduction:**

Understanding changes in opioid overdose-related emergency department (ED) visits and ED-based opioid use disorder (OUD) treatment post-coronavirus disease 2019 (COVID-19) pandemic can inform ongoing efforts to address the opioid crisis. We aimed to examine trends in opioid overdose-related ED visits, ED-based medication for OUD (MOUD) treatment, and appendicitis-related ED visits (as a control) before and after the initial COVID-19 peak in April 2020.

**Methods:**

We conducted an interrupted time series analysis of monthly ED visits from January 2017–December 2022 at three hospitals in California. We modeled pre- and post-COVID-19 visit trends and the change in trend from pre- to post-COVID-19 peak using linear regression controlling for study site. Our primary outcome included monthly rates of opioid overdose-related ED visits, with monthly rates of ED visits with MOUD treatment as a secondary outcome. Appendicitis-related visits served as a control for temporal trends.

**Results:**

Of the 781,488 ED visits across the entire study period, there were 2,536 (0.32%) opioid overdose-related visits, 9,755 (1.25%) MOUD treatment visits, and 1,123 (0.14%) appendicitis-related visits. Pre-pandemic, monthly increases were observed in opioid overdose-related visits (6.7 visits/10,000 per month, 95% confidence interval [CI] 2.6–10.8, *P* = .001), MOUD-positive visits (34.9 visits/10,000 per month, 95% CI, 26.0–43.7, *P* < .001), and appendicitis visits (2.5 visits/10,000 per month, 95% CI, 1.2–3.9, *P* < .001). After April 2020, only MOUD-positive visits showed an immediate (level change) increase (49 visits/10,000 or 34% of April 2020 projected pre-COVID-19 visit rates, 95% CI, 26.1–71.9, *P* < .001), with opioid overdose-related visits subsequently declining (−4.6 visits/10,000 per month or −11% of April 2020 projected pre-COVID-19 visit rates, 95% CI, −7.2 to −2.0, *P* = .001). Across the entire pre- to post-COVID-19 period, significant decreases in overall visit trends were observed across all visit types, greatest for MOUD-positive visits (−39.2 visits/10,000 per month, or −27% of April 2020 projected pre-COVID-19 visit rates, 95% CI, −51.5 to −26.8, *P* < .001), followed by opioid overdose visits (−11.3 visits/10,000 per month, or −27% of April 2020 projected pre-COVID-19 visit rates, 95% CI, −16.1 to −6.5, *P* < .001) and appendicitis visits (−4.2 visits/10,000 per month, or −23% of April 2020 projected pre-COVID-19 visit rates, 95% CI, −7.2 to −1.2, *P* = .01).

**Conclusion:**

From pre- to post-initial COVID-19 peak, absolute ED-based MOUD treatment trends declined over three times faster than those of opioid overdose-related ED visits (although percentage changes relative to expected April 2020 rates were both −27%). These findings may reflect reduced perceived urgency as overdose presentations decreased and a shift away from crisis-driven implementation, underscoring the need for intentional integration of MOUD into routine ED practice to sustain treatment capacity.

## INTRODUCTION

The coronavirus disease 2019 (COVID-19) pandemic disrupted many aspects of society in the United States, necessitating social isolation and the shutdown of businesses, schools, and other public entities. These disruptions, combined with COVID-19 itself, not only negatively impacted the U.S. population’s physical^1^ and mental health^2^,^3^ but also reduced access to many social services.^4^

People with opioid use disorder (OUD) were particularly vulnerable to disruptions in access to addiction treatment and social support during the COVID-19 pandemic.^5^ While emergency department (ED) visits decreased overall during the peak of the pandemic,^6^ there was an increase in opioid overdose-related visits.^5^,^7^ Several factors likely contributed to this trend, including reduced access to in-person addiction services, heightened pandemic-related stressors (eg, social isolation, financial instability), and worsening mental health leading to increased substance use.^8^ These disruptions spurred innovative strategies to expand treatment access, including removal of prescribing restrictions and rapid expansion of telehealth-based addiction care. Around the same time, EDs—recognized as the safety net for patients without access to outpatient care—saw increased efforts to initiate medications for OUD (MOUD), such as methadone and buprenorphine.^9^

While trends in opioid overdose-related ED visits before and during the COVID-19 pandemic are well described, less is known about post-pandemic changes in overdose-related ED visits and corresponding MOUD treatment rates following these policy and practice changes. Understanding how opioid overdose-related ED visits and ED-based MOUD treatment have changed after the pandemic can offer valuable insights into the impact and sustainability of OUD treatment strategies, while also helping to identify persistent gaps in care that require targeted intervention. We sought to examine trends in opioid overdose-related ED visits and EDbased MOUD treatment around the first COVID19 peak. Prior studies have demonstrated substantial pandemic-associated declines in ED presentations for time-sensitive emergencies, including acute myocardial infarction,^10^ suggesting that utilization changes were diagnosis-specific. We used appendicitis as a comparator condition, as several U.S. studies found stable presentation volumes during the peri-pandemic period.^11^,^12^

## METHODS

Using electronic health record data at the encounter level, we performed an interrupted time series analysis examining all monthly ED visits from January 2017–December 2022 at three hospitals (two academic; one county) in California, with April 2020 (first COVID-19 peak) as the interruption. Selecting December 2022 as the study end date avoided confounding from national policy changes implemented in 2023 (e.g., elimination of the Drug Addiction Treatment Act of 2000 (DATA 2000) Xwaiver). Our primary outcome was opioid overdose-related ED visits, defined by *International Classification of Diseases, 10th Revision, Clinical Modification (ICD-10-CM)* codes T40.0-T40.4 and T40.6. We calculated monthly visit rates by dividing overdose-related visits by total ED visits to account for secular trends affecting total ED volume. We examined appendicitis-related visits as a control for temporal trends.

**Population Health Research Capsule**

What do we already know about this issue?

*While overall emergency department (ED) visits declined during COVID-19, opioid overdose-related ED visits increased*.

What was the research question?

*How did ED visits for opioid overdose and ED-based opioid use disorder (OUD) treatment trends change after the first COVID-19 peak?*

What was the major finding of the study?

*Overdose visits declined −11.3 vs −39.2 visits/10k/month for OUD treatment (95% CIs, −16.1 to −6.5; −51.5 to −26.8)*.

How does this improve population health?

*Although overdose visits declined post-COVID-19, the faster drop in ED-based OUD treatment underscores the need to sustain treatment capacity*.

To assess ED-based treatment for OUD, we classified ED visits as positive for MOUD if methadone or buprenorphine was ordered during the ED visit. Naltrexone was excluded as it is generally considered a second-line therapy for OUD. Because our goal was to measure system-level volumes, we counted each visit type (ie, opioid overdose, appendicitis, MOUD) separately and did not quantify overlap. For example, a single encounter could contribute to both overdose-related visits and MOUD treatment visits. We also did not distinguish MOUD given to mitigate withdrawal from MOUD initiated for OUD, as in ED practice, MOUD are typically given in the presence of withdrawal symptoms and, by design, serve as treatment for OUD.

We used linear regression controlling for study site with robust standard errors, establishing the underlying monthly visit trends and determining changes post-interruption.^13^ Excluding April 2020 data as a washout period, we estimated the level change in May 2020 using the interruption period variable and a slope change using the time after the interruption variable to assess trends over time. Autocorrelation plots showed no consistent correlation between time points, so adjustment was deemed unnecessary. We performed three sensitivity analyses: 1) adjusting for seasonality by including yearly quarters in our model; 2) aggregating data by yearly quarters instead of monthly intervals; and 3) including April 2020 data in the exposure period. To preserve the full effect of the COVID-19 peak, we excluded patient- and encounter-specific variables potentially influenced by the pandemic.

We conducted analyses using R version 4.42 (The R Foundation for Statistical Computing, Vienna, Austria), The University of California, San Francisco Institutional Review Board approved this study, and we followed Strengthening the Reporting of Observational Studies in Epidemiology (STROBE) guidelines.

## RESULTS

From January 1, 2017– December 31, 2022, we analyzed 781,488 ED visits (excluding 7,625 visits from April 2020 as part of the washout period), with 2,536 (0.32%) representing opioid overdose-related ED visits; 1,123 (0.14%) appendicitis-related ED visits, and 9,755 (1.25%) ED visits during which MOUD were ordered. Total monthly visits in either study site ranged between 3,750–7,451 during the study period. The overall yearly ED visits by visit type are presented in Supplement 1.

Prior to the initial COVID-19 peak, opioid overdose-related ED visits had been increasing by 6.7 visits/10,000 per month (95% CI, 2.6–10.8), with a steeper increase in MOUD-positive visits (34.9 visits/10,000 per month, 95% CI, 26.0–43.7). Appendicitis-related visits were increasing by 2.5 visits/10,000 per month (95% CI, 1.2–3.9). After April 2020, there was a sharp rise in MOUD-positive visits (49 visits/10,000 per month or 34% of April 2020 projected pre-COVID-19 visit rates, 95% CI, 26.1–71.9) with no significant level change in either opioid overdose- (1.2 visits/10,000 per month, 95% CI, −8.0 to 10.3) or appendicitis-related ED visits (−1.4 visits/10,000 per month, 95% CI, −7.1to 4.3). We subsequently observed a significant decrease in opioid overdose-related ED visits by 4.6 visits/10,000 per month (or −11% of April 2020 projected pre-COVID-19 visit rates, 95% CI, −7.2 to −2.0), and non-significant decreases in MOUD-positive visits (−4.3 visits/10,000 per month, 95% CI, −12.9 to 4.3) and appendicitis-related visits (−1.7 visits/10,000 per month, 95% CI, −4.4 to 1.0) (Figure).

All visit types showed significant decreases in slope from pre- to post-COVID-19, with appendicitis-related visits changing the least (−4.2 visits/10,000 per month or −23% of April 2020 projected pre-COVID-19 visit rates, 95% CI, −7.2 to −1.2), followed by overdose-related visits (−11.3 visits/10,000 per month or −27% of April 2020 projected pre-COVID-19 visit rates, 95% CI, −16.1 to −6.5), and MOUD-positive visits (−39.2 visits/10,000 per month or −27% of April 2020 projected pre-COVID-19 visit rates, 95% CI, −51.5 to −26.8) (Table). Sensitivity analyses confirmed our primary findings, with the following exceptions: 1) in the quarterly analysis, the pre- to post-April 2020 slope change for appendicitis visits lost statistical significance; 2) when April 2020 data was included, the post-April 2020 decrease in opioid overdose-related ED visits lost statistical significance (−3.1 visits/10,000 per month, 95% CI, −6.5 to 0.3).

## DISCUSSION

In this interrupted time series analysis of 781,488 ED visits at three hospitals, we found that all visit types were increasing before the pandemic. Immediately following the initial COVID-19 peak, only MOUD-positive visits showed a significant increase in level change. The subsequent decline in opioid overdose-related visits occurred nearly three times faster than that of appendicitis-related visits. All visit types experienced significantly decreased visit trends (ie, negative slope changes) from pre- to post-COVID-19 peak, with MOUD visits declining the most, followed by overdose-related visits, and appendicitis-related visits.

Although our study period centered on the peri-pandemic era, it coincided with major regulatory changes and care-delivery innovations, serving as a natural experiment in how system-level change coincided with trends in ED presentation and treatment. The level changes in opioid overdose- and appendicitis-related ED visits after April 2020 align with prior research showing increased overdose-related visits early in the pandemic, despite an overall decline in ED use.^5^ To address rising overdose rates, California expanded its ED Bridge Program between 2018–2020, partnering with 52 hospitals—including two of our three study sites—to provide low-threshold buprenorphine treatment and connect patients to outpatient care and harm reduction services.^14^,^15^ While our data show an initial increase in ED-based MOUD treatment pre-pandemic, the overall decrease in MOUD-positive visits from pre- to post-April 2020 was more than three times greater than the decline in opioid overdose-related visits.

This pattern—an early rise followed by lack of sustained growth—highlights a continuing implementation challenge: expanding ED MOUD initiation capacity is achievable, but maintaining and scaling it requires deliberate sustainment infrastructure and policy support. Sustained integration of MOUD into ED practice will likely require addressing ED-specific barriers, including time constraints, competing clinical demands, and workflow disruptions that limit opportunities for treatment initiation.

The observed post-pandemic decrease in opioid overdose-related ED visits may reflect multiple factors. While expanded access to addiction treatment services, including telehealth options and increased MOUD availability, may have played a role, the declining trend could also reflect the resolution of pandemic-related stressors. These included the lifting of social isolation requirements, economic recovery, and restored access to in-person mental health and community support services. As health systems moved into 2023, several concurrent developments may have influenced both overdose presentations and OUD treatment. Removal of the DATA 2000 Xwaiver and implementation of overthecounter naloxone broadened access, yet ED capacity strains due to inpatient boarding and staffing shortages may have reduced capacity to consistently initiate MOUD. Additionally, prehospital naloxone treatment may have shifted some nonfatal overdoses away from the ED. Future work should extend these analyses beyond 2022 to evaluate the impact of these changes on overdose presentations and EDbased MOUD treatment.

## LIMITATIONS

Our retrospective analysis covered the peri-pandemic period through 2022. Although practice patterns and access to addiction services have since evolved, this period functioned as a system-stress natural experiment that yields important lessons about ED MOUD adoption, utilization trends, and sustainment challenges. Our decision not to include patient- or encounter-level covariates in the interrupted time series models to preserve the effect of the initial COVID-19 peak may have obscured important subgroup interactions that warrant future study. Our aggregated, encounter-level time-series approach showed visit-to-visit variability around trend lines, indicating heterogeneity across patients, clinicians, and sites; accordingly, the reported slopes should be interpreted as population-level directional estimates rather than uniform effects at the encounter level.

We could not identify the drivers of observed trends, which may have been influenced by local and regional factors. Our reliance on *ICD-10-CM* codes may have underestimated the true prevalence of overdose- and appendicitis-related visits due to coding variability and misclassification, although the presence of significant trends despite this limitation lends credibility to our findings. We measured MOUD orders rather than administrations to capture treatment efforts, acknowledging that not all orders result in medication administration. Finally, results from three Northern California hospitals may not generalize to other settings with different policies, resources, or addiction care infrastructures.

## CONCLUSION

In an interrupted time series analysis of ED visits before and after the initial COVID-19 peak, absolute ED-based MOUD treatment trends declined over three times faster than those of opioid overdose-related ED visits (although percentage changes relative to expected April 2020 rates were both −27%). The steeper decline in MOUD initiation may reflect reduced perceived urgency to initiate treatment as overdose presentations decreased, compounded by post-pandemic ED capacity constraints and workflow pressures. Sustaining ED-based MOUD likely requires intentional integration into routine practice rather than reliance on crisis-driven implementation.

## Supplementary Information



## Figures and Tables

**Figure:**
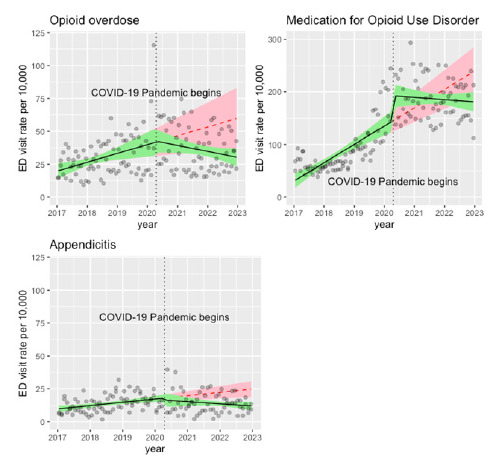
An interrupted time series analysis of the monthly rate of ED visits, in a study examining trends in opioid overdose-related ED visits (2017–2022). Shading indicates 95% CI; dashed lines, the forecasted ED visits; solid lines, the observed trends in ED visits; and dotted line, the initial COVID-19 peak. *ED*, emergency department.

**Table:** Interrupted time series models for emergency department visits^a^ by visit type before and after the initial COVID-19 peak, at three hospitals in California with a total of 781,488 visits (2017–2022).

	Slope, Pre-COVID A (95% CI) *P*-value	Level Change X (95% CI) *P*-value	Slope, Post-COVID B (95% CI) *P*-value	Slope Change B-A (95% CI) *P*-value
Opioid Overdose	6.7 (2.6, 10.8) *P* = .001	1.2 (−8.0, 10.3) *P* = .80	−4.6 (−7.2, −2.0) *P* = .001	−11.3 (−16.1, −6.5) *P* < .001
MOUD^b^	34.9 (26.0, 43.7) *P* < .001	49.0 (26.1, 71.9) *P* < .001	−4.3 (−12.9, 4.3) *P* = .33	−39.2 (−51.5, −26.8) *P* < .001
Appendicitis	2.5 (1.2, 3.9) *P* < .001	−1.4 (−7.1, 4.3) *P* = .63	−1.7 (−4.4, 1.0) *P* = .22	−4.2 (−7.2, −1.2) *P* = .01

a Emergency department visits per 10,000 per month.

b Emergency department visits per 10,000 per month during which either methadone or buprenorphine were ordered

MOUD, medication for opioiod use disorder.
