# Supplementary material for: Opioid Overdose-Related Emergency Department Visits Pre- and Post-COVID-19
Source: West J Emerg Med. 2026 Jun 10;27(4):875–9. doi: 10.5811/westjem.53227 (PMC13436652; doi:10.5811/westjem.53227)
Supplement: Supplementary file 1 [file wjem-27-875-s001.docx]

**Supplement 1.** Yearly emergency department (ED) visit counts and rates by visit type (2017-2022)

|  | Total counts (rates) per 10,000 emergency department visits | | |
| --- | --- | --- | --- |
| Year | Opioid Overdose  N = 2,536 | MOUD^a^  N=9,755 | Appendicitis  N = 1,123 |
| 2017 | 345 (0.24%) | 849 (0.59%) | 155 (0.11%) |
| 2018 | 452 (0.31%) | 948 (0.64%) | 213 (0.14%) |
| 2019 | 492 (0.35%) | 1,621 (1.12%) | 252 (0.18%) |
| 2020 | 450 (0.43%) | 1,943 (1.85%) | 162 (0.15%) |
| 2021 | 402 (0.34%) | 2,166 (1.81%) | 168 (0.14%) |
| 2022 | 395 (0.32%) | 2,228 (1.81%) | 173 (0.14%) |
| Total | 2,536 (0.32%) | 9,755 (1.25%) | 1,123 (0.14%) |

^a^Emergency department visits during which either methadone or buprenorphine were ordered

In a study examining trends in opioid overdose-related ED visits and ED‑based MOUD treatment around the first COVID‑19 peak, with ED visits for appendicitis as a control, Supplement 1 shows yearly ED visits by visit type from January 1, 2017 through December 31, 2022.

*MOUD*, medication for opioid use disorder; *COVID-19*, Coronavirus Disease of 2019
